# Selective Upcycling of Waste PET into Di(2-ethylhexyl) Terephthalate via a Hybrid Organometallic Catalytic Strategy for PVC Plasticizer Applications

**DOI:** 10.3390/polym18192381

**Published:** 2026-09-29

**Authors:** Ahmet Erdem, Rukiye Gunay, Didem Aksoy, Melike Ozkan, Fahanwi Asabuwa Ngwabebhoh

**Affiliations:** 1 Department of Biomedical Engineering, Faculty of Technology, Kocaeli University, Kocaeli 41001, Turkey; 2 Department of Polymer Science and Engineering, Institute of Science, Kocaeli University, Kocaeli 41001, Turkey; 3 Plastay Kimya Sanayi ve Ticaret A.Ş., Gebze Organized Industrial Zone, Kocaeli 41400, Turkey; 4 Department of Chemistry, Faculty of Science and Arts, Kocaeli University, Kocaeli 41001, Turkey

**Keywords:** waste PET, DOTP, hybrid organometallic catalyst, chemical recycling, PVC plasticizer

## Abstract

The selective conversion of post-consumer polyethylene terephthalate (PET) into value-added plasticizers offers a promising pathway for advancing polymer circularity within the scope of the circular economy approach. Herein, waste PET bottles were directly upcycled into di(2-ethylhexyl) terephthalate (DOTP) via degradative alcoholysis/transesterification with 2-ethylhexanol using four organometallic catalyst systems: butylstannic acid (F), monobutyltin tris(2-ethylhexanoate) (TK), titanium tetraisopropoxide (T), and a hybrid organotin–titanium system (TKT). PET conversion ranged from 84 to 94%, with the T catalyst affording the highest conversion (94%). Notably, the hybrid TKT catalyst provided the most favorable overall performance, achieving an 81% isolated yield and 85.4% selectivity. GC-FID analysis revealed that DOTP-TKT achieved a 92.0% chromatographic area, while GPC confirmed that this product had the lowest proportion of residual oligomers (15.13%) among all PET-derived samples. FTIR and ^1^H-NMR spectroscopy further confirmed efficient PET-to-DOTP transformation, with DOTP-TKT displaying the closest structural correspondence to commercial DOTP. Amongst the PET-derived products, DOTP-TKT exhibited the most favorable thermal behavior, with a principal maximum degradation temperature (T_max_) of approximately 283.5 °C. When incorporated into PVC, PET-derived DOTP-TKT showed enhanced tensile strength, elastic modulus, and elongation at break compared with commercial DOTP. These findings demonstrate that the combined Ti–Sn catalyst system simultaneously promotes selective PET depolymerization and product formation while limiting oligomeric residues, providing an effective route for converting post-consumer PET into functional, high-value PVC plasticizers within a circular polymer economy.

## 1. Introduction

Globally, polyethylene terephthalate (PET) is among the most extensively utilized thermoplastics due to its excellent mechanical strength, chemical resistance, transparency, and processability, enabling its widespread application in packaging, textiles, and a broad range of consumer products [1,2]. However, the continuous growth in PET consumption has generated substantial quantities of persistent post-consumer waste, intensifying concerns over resource depletion and environmental pollution. Consequently, transitioning from conventional waste disposal toward circular material management has become an important objective of sustainable chemistry and the circular economy [3,4]. Amongst these approaches, mechanical recycling remains the predominant route for PET recovery but is constrained by progressive thermo-mechanical degradation, contamination, and the deterioration of material properties over repeated processing cycles. Such limitations frequently result in downcycled products with reduced economic value [5,6,7]. Chemical recycling and upcycling provide an alternative by cleaving PET ester linkages to recover monomers or transform the polymer directly into value-added chemical derivatives [6,8,9]. Particularly attractive is the conversion of waste PET into plasticizers, which simultaneously diverts persistent polymer waste from disposal and replaces virgin petrochemical feedstocks with secondary carbon resources [10,11].

Plasticizers are essential additives for improving the flexibility, processability, and service performance of poly(vinyl chloride) (PVC) in applications ranging from construction and cables to textiles and biomedical products [12,13]. Increasing concerns regarding the potential adverse effects of conventional ortho-phthalate plasticizers have stimulated demand for alternative formulations. Di(2-ethylhexyl) terephthalate (DOTP), a non-ortho-phthalate plasticizer, has consequently emerged as an important alternative because of its favorable performance and low toxicological profile [14]. The direct alcoholysis/transesterification of post-consumer PET with 2-ethylhexanol (2-EH) represents an attractive waste-to-product pathway in which the terephthalate framework of PET is retained within a commercially relevant plasticizer. However, catalyst selection is central to this transformation because PET conversion, the reaction rate, DOTP selectivity, oligomer formation, and final product quality are strongly catalyst-dependent. Conventional systems including zinc salts, acids, ionic liquids, and organometallic compounds have been investigated [14,15,16,17], with titanium-based catalysts receiving considerable attention because of their Lewis acidity and high transesterification activity [18]. For example, a previous study using tetrabutyl titanate as a catalyst afforded a DOTP yield of 78.10% using a catalyst/PET mass ratio of 0.5% and a 2-EH/PET molar ratio of 4 [19], while in a recent report, the use of zinc acetate and tetrabutyl titanate as catalysts enabled approximately 85% yield in ionic-liquid-assisted PET conversion [20]. More recently, polynuclear titanium catalysts demonstrated exceptionally high activity in PET methanolysis through the cooperative activation of ester and alcohol functionalities [21]. Moreover, titanium catalysts may promote undesirable oligomer formation, discoloration, or catalyst-derived impurities under demanding reaction conditions [22]. As such, organotin catalysts offer complementary characteristics, particularly high selectivity and thermal stability. In a recent study, monobutyltin tris(2-ethylhexanoate) was reported to produce a 61.1% DOTP yield after 3 h [20], while butylstannic acid, containing catalytically active Sn–O–Sn functionalities, demonstrated activity toward PET alcoholysis and polyester depolymerization [23]. However, organotin systems can exhibit comparatively slow initial reaction kinetics, potentially extending processing time and increasing energy demand [24]. Thus, titanium and organotin catalysts possess distinct advantages but also catalyst-specific limitations that restrict optimization based on either system alone.

Despite these advances, systematic comparisons of titanium- and organotin-based catalysts under identical PET alcoholysis conditions remain limited, and the potential cooperative effect of combining these catalyst families for direct PET-to-DOTP upcycling has not been sufficiently established. The present study addresses this gap by comparatively evaluating butylstannic acid, tetraisopropyl titanate, and monobutyltin tris(2-ethylhexanoate), alongside a hybrid titanium–organotin catalytic strategy, under equivalent processing conditions. The key novelty of this study lies in evaluating a hybrid titanium–organotin catalytic system as a strategy to balance PET conversion with the selective formation of the targeted ester product while limiting the persistence of oligomeric byproducts. Product composition, molecular structure, purity, and thermal properties were systematically elucidated using GC-FID, GPC, FTIR, ^1^H-NMR, and TGA. Importantly, PET-derived DOTP was further incorporated into PVC and benchmarked against commercial DOTP through mechanical testing, thereby linking catalyst chemistry and product quality directly to end-use plasticizer performance. This integrated catalyst-to-application approach provides a pathway for converting post-consumer PET into a value-added functional additive within a circular polymer framework.

## 2. Materials and Methods

### 2.1. Materials

**Figure 1:**
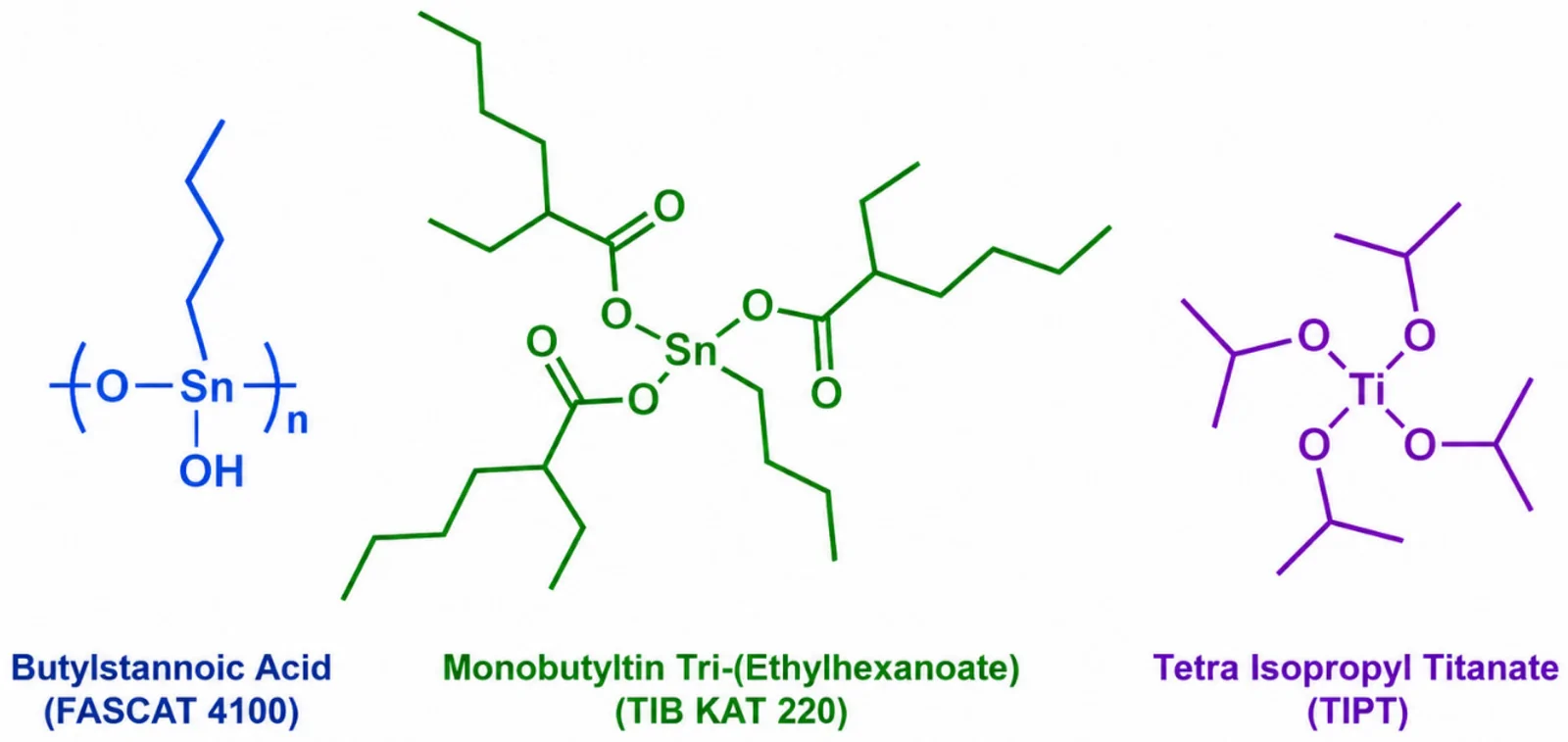
Molecular structures of catalysts used in DOTP synthesis from waste PET: butyl stannic acid (FASCAT 4100-F), monobutyltin tris(2-ethylhexanoate) (TIB KAT 220-TK), and tetra isopropyl titanate (TIPT-T).

Post-consumer polyethylene terephthalate (PET) bottles were used as the primary feedstock. The bottles were cleaned, dried, and mechanically cut into flakes with an approximate size of 5–10 mm prior to use. 2-Ethylhexan-1-ol (2-EH, ≥99% purity; BASF SE, Ludwigshafen, Germany) was used as the alcoholysis/transesterification reagent for the synthesis of di(2-ethylhexyl) terephthalate (DOTP). Three commercial organometallic catalysts were employed: butylstannic acid (F; FASCAT^®^ 4100, PMC Organometallix, Inc., Carrollton, KY, USA), monobutyltin tris(2-ethylhexanoate) (TK; TIB KAT^®^ 220, TIB Chemicals AG, Mannheim, Germany), and titanium (IV) isopropoxide/tetraisopropyl titanate (T; TIPT, Adakem Kimya Ltd., Istanbul, Turkey) (Figure 1). These catalysts were used individually and, where applicable, in combination to formulate the hybrid titanium–organotin catalytic system. For the preparation and performance evaluation of the plasticized PVC formulations, suspension-grade poly(vinyl chloride) (PVC; K-value = 70), a liquid calcium/zinc (Ca/Zn)-based thermal stabilizer, and commercial-grade DOTP (Plastay Kimya Sanayi ve Ticaret A.Ş., Gebze, Kocaeli, Turkey) were used. The commercial DOTP served as the reference plasticizer for comparison with the DOTP synthesized from post-consumer PET. Unless otherwise stated, all chemicals and reagents were used as received without further purification.

### 2.2. DOTP Synthesis from Waste PET

**Figure 2:**
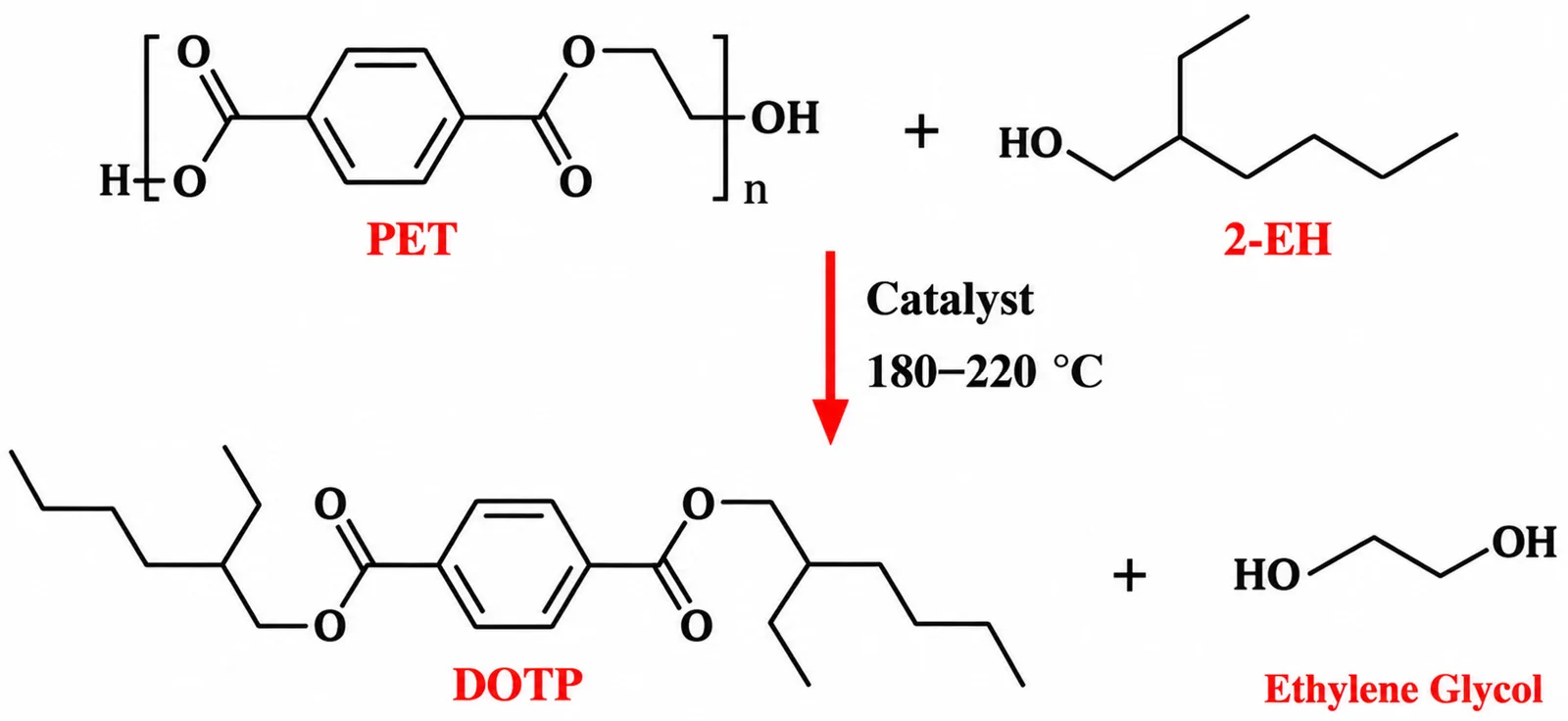
Schematic reaction of conversion of waste PET to DOTP by alcoholysis reaction with 2-ethylhexanol (2-EH).

Recycled DOTP was synthesized from post-consumer PET through catalytic alcoholysis/transesterification with 2-ethylhexanol (2-EH). The reaction was conducted in a four-neck glass reactor equipped with a mechanical stirrer, thermometer, reflux condenser, and Dean–Stark separator. For each experiment, PET flakes (192 g, corresponding to 1 mol of PET repeating units) and 2-EH (4 mol) were charged into the reactor, maintaining a PET:2-EH molar ratio of 1:4 in accordance with previously reported conditions [17,25]. After the reaction mixture reached the target temperature, the respective catalyst was introduced at 3 wt% relative to the initial PET mass. Butylstannic acid (F), monobutyltin tris(2-ethylhexanoate) (TK), and titanium(IV) isopropoxide (T) were evaluated individually. For the hybrid titanium–organotin system (TKT), the total catalyst loading was maintained at 3 wt%, comprising 1.5 wt% T and 1.5 wt% TK (Table 1). This formulation maintained the same overall catalyst loading as the individual catalyst systems and enabled the evaluation of the performance of the combined Ti–Sn catalyst system under otherwise identical reaction conditions. The reaction mixture was maintained at 180–220 °C for 4 h under continuous mechanical stirring. Ethylene glycol (EG), generated through the cleavage and transesterification of the PET ester bonds, was continuously separated from the reaction medium to shift the equilibrium toward DOTP formation. Following reaction completion, unreacted excess 2-EH was recovered by vacuum distillation. The resulting crude DOTP was subsequently washed with deionized water to remove residual catalyst and water-soluble impurities, followed by filtration to obtain the purified product. The proposed PET-to-DOTP conversion pathway is illustrated in Figure 2.

The isolated DOTP yield and corrected DOTP yield were calculated gravimetrically and chromatographically from the mass of the recovered purified product relative to the theoretical DOTP mass according to Equations (1) and (2) [26]:

(1) $$Y_{iso}\left(\%\right)=\frac{M_{f}}{M_{t}}\times 100$$

(2) $$Y_{corr}\left(\%\right)=Y_{iso}\times \frac{A_{GC}}{100}$$

where *Y_iso_* is the isolated DOTP yield (%), *Y_corr_* is the corrected DOTP yield (%), *M_f_* is the mass of the recovered purified product after reaction and purification (g), *M_t_* is the theoretical mass of DOTP expected from the complete conversion of the initial PET feedstock (g), and *A_GC_* is the relative DOTP content (%) determined independently by GC-FID. PET conversion was calculated from the amount of residual unreacted PET according to Equation (3) [27]:

(3) $$C_{PET}\left(\%\right)=\frac{M_{s}-M_{r}}{M_{s}}\times 100$$

where *C_PET_* is the PET conversion (%), *M_s_* is the initial mass of PET introduced into the reaction (g), and *M_r_* is the mass of residual unreacted PET recovered after the reaction (g). For the accurate gravimetric determination of residual PET, the recovered solid residue was washed with ethanol to remove adhering reaction components and subsequently dried at 100 °C to constant mass before weighing. This procedure was applied identically to all catalyst systems. An apparent selectivity toward the isolated DOTP-rich product was subsequently derived from the relationship between corrected product yield and PET conversion according to Equation (4) [16]:

(4) $$S_{DOTP}(\%)=\frac{Y_{corr}}{C_{PET}}\times 100$$

where *S_DOTP_* is the apparent or derived selectivity (%), *Y_corr_* is the corrected DOTP yield (%), and *C_PET_* is the PET conversion (%). Accordingly, the reported selectivity represents a derived process performance parameter rather than an independently measured product selectivity obtained from the complete chromatographic speciation of all reaction products. All the catalyst systems were evaluated under an identical PET/2-EH ratio, total catalyst loading, reaction temperature, and reaction time to enable the direct comparison of their effects on PET conversion, product recovery, and apparent selectivity.

### 2.3. Characterization of Recycled DOTP

The chemical structure, chromatographic composition, molecular size distribution, thermal stability, and physicochemical properties of the PET-derived DOTP samples synthesized using the different catalyst systems were systematically evaluated using complementary analytical techniques. Commercial DOTP was analyzed under identical or comparable conditions, where applicable, and used as a reference material. The characterization procedures are described separately below to facilitate the reproducibility and interpretation of the analytical results.

#### 2.3.1. Fourier Transform Infrared Spectroscopy

Fourier transform infrared (FTIR) spectroscopy was employed to confirm the chemical transformation of post-consumer PET into di(2-ethylhexyl) terephthalate and to identify the characteristic functional groups of the synthesized products. The FTIR spectra of commercial DOTP and the PET-derived products (DOTP-F, DOTP-T, DOTP-TK, and DOTP-TKT) were recorded over the wavenumber range of 4000–600 cm^−1^, using an ATR accessory of the Spectrum 100 FTIR spectrometer (PerkinElmer Inc., Waltham, MA, USA).

#### 2.3.2. Proton Nuclear Magnetic Resonance Spectroscopy

The molecular structures of the synthesized PET-derived DOTP products were further characterized by proton nuclear magnetic resonance (^1^H-NMR) spectroscopy. Spectra were acquired using an AVANCE III 400 MHz NMR spectrometer (Bruker BioSpin GmbH, Rheinstetten, Germany) operating at 400 MHz. Samples were dissolved in deuterated chloroform (CDCl_3_) prior to analysis. The spectra of the PET-derived products were compared with that of commercial DOTP to evaluate structural correspondence and to qualitatively identify additional resonances potentially associated with residual reaction-derived species.

#### 2.3.3. Gas Chromatography–Flame Ionization Detection Analysis

Gas chromatography coupled with flame ionization detection (GC-FID) was used to evaluate the chromatographic composition of commercial DOTP and the products obtained from PET alcoholysis. Analyses were performed using a GC-FID system (Bruker Corporation, Billerica, MA, USA) equipped with a Column Restek/Rxi-5ms capillary column. Nitrogen was used as the carrier gas at a flow rate of 1 mL min^−1^. The injector and FID temperatures were maintained at 300 °C. Samples were injected at a volume of 1 µL using a split ratio of 1:80. The oven temperature program was followed by heating at 10 °C min^−1^ to 300 °C. GC-FID was employed to comparatively evaluate the chromatographic composition of the recovered products. The principal DOTP peak was identified by comparison with commercial DOTP, and the relative DOTP content was determined according to the applied chromatographic procedure. Minor chromatographic signals were evaluated separately and, in the absence of confirmation by authentic standards or GC-MS, were not assigned definitively to specific reaction intermediates.

#### 2.3.4. Gel Permeation Chromatography

Gel permeation chromatography (GPC) was employed as a comparative technique to assess the relative occurrence of higher-molecular-weight species and residual oligomeric fractions in the PET-derived products. Analyses were performed using a Viscotek GPCmax system equipped with a VE 3580 refractive index detector and Viscotek LT4000L columns (Viscotek Corporation, Houston, TX, USA), utilizing tetrahydrofuran (THF) as the mobile phase at 35 °C with a flow rate of 1.0 mL min^−1^. Samples were dissolved at a concentration of 12 mg mL^−1^, and an injection volume of 100 µL was applied. The system was calibrated using nine monodisperse polystyrene standards spanning a nominal molecular weight range of 1.2 to 400 kDa, with data processed using OmniSEC software (version 5.12). Because the molecular weight of DOTP (~390 g/mol) lies below the lower limit of the calibration range, GPC was not utilized for absolute molecular weight determination or quantitative purity calculation. Instead, the resulting chromatograms were interpreted qualitatively and comparatively to evaluate variations in the relative abundance of earlier-eluting, higher-hydrodynamic-volume species.

#### 2.3.5. Thermogravimetric Analysis

The thermal stability and degradation behavior of commercial DOTP and the PET-derived products were investigated by thermogravimetric analysis (TA) using a TA 5500 analyzer (TA Instruments, New Castle, DE, USA). Approximately 20 mg of sample was placed in alumina pans and heated from 25 °C to 600 °C at a heating rate of 10 °C min^−1^ under a nitrogen atmosphere. Both thermogravimetric (TGA) and derivative thermogravimetric (DTG) curves were evaluated. The onset of thermal degradation (T_onset_) and the temperature corresponding to the maximum mass loss rate (T_max_) were distinguished during data interpretation. Residual mass at elevated temperature was additionally recorded to assess the presence of non-volatile components.

#### 2.3.6. Physical Characterization

The physicochemical properties of the PET-derived DOTP products were evaluated in terms of acid value, dynamic viscosity, density, and color and compared with those of commercial DOTP. The acid value was determined by acid–base titration and expressed as milligrams of potassium hydroxide required to neutralize the acidic constituents present in 1 g of sample (mg KOH g^−1^). Briefly, 1 g of each sample was dissolved in Toluene/isopropyl alcohol and titrated with standardized 0.01 M KOH solution using phenolphthalein. The acid value was calculated using Equation (5):

(5) $$AN=\frac{V\times C\times 56.1}{m}$$

where *AN* is the acid number (mg KOH g^−1^), *V* is the volume of KOH solution consumed during titration (mL), *C* is the concentration of the KOH solution (mol L^−1^), 56.1 is the molar mass of KOH (g mol^−1^), and *m* is the sample mass (g).

Dynamic viscosity was measured at 20 °C using an IKA ROTAVISC rotational viscometer (IKA-Werke GmbH & Co. KG, Staufen, Germany) in accordance with ASTM D1045 [28]. Prior to measurement, the samples were equilibrated at the measurement temperature for 15 min. Viscosity measurements were performed using an Ultra-Low Adapter spindle at a rotational speed of 60 rpm. At least three measurements were obtained for each formulation, and the results were expressed in centipoise (cP). Density was determined at 20 °C according to ASTM D1045 using a pycnometer and expressed as g cm^−3^. For the pycnometric method, a calibrated 10 mL pycnometer was filled with the temperature-equilibrated sample, and density was calculated from the measured sample mass and calibrated pycnometer volume. Furthermore, the color of the commercial and PET-derived DOTP samples was determined according to ASTM D1209 [28] using the platinum–cobalt (Pt–Co) color scale. Measurements were performed using a CS-826 desktop spectrophotometer (CHN Spec Technology Co., Ltd., Hangzhou, China) after appropriate instrument calibration, and the results were expressed as Pt–Co color units. Lower Pt–Co values indicate the lower coloration of the plasticizer. Unless otherwise stated, physicochemical measurements were performed in triplicate.

### 2.4. Preparation and Mechanical Testing of PVC Blends Plasticized with PET-Derived DOTP

The plasticization performance of the PET-derived DOTP product exhibiting the most favorable overall physicochemical characteristics (DOTP-TKT) was evaluated in flexible poly(vinyl chloride) (PVC) formulations and compared with that of commercial DOTP. To ensure direct comparison between the two plasticizers, identical PVC formulations and processing conditions were employed.

#### 2.4.1. Preparation of Plasticized PVC Formulations

PVC compounds were formulated using 100 parts per hundred resin (phr) of suspension-grade PVC (K-value = 70), 50 phr of either commercial DOTP or PET-derived DOTP-TKT, and 1 phr of a liquid Ca/Zn-based thermal stabilizer. No other additives were incorporated unless otherwise stated. The PVC resin, plasticizer, and thermal stabilizer were initially premixed for 15 min using a mixer to facilitate the uniform distribution of the liquid components throughout the PVC powder. The resulting mixtures were subsequently processed using a laboratory-scale twin-screw extruder with a screw diameter of 11 mm, a length-to-diameter ratio (L/D) of 40:1, and a screw rotation speed of 40 rpm. The temperature profile along the extruder barrel was maintained within the range of 170–180 °C, with individual heating zones set at Zone 1: 150 °C/Zone 2: 165 °C/Zone 3: 175 °C/die: 180 °C, where applicable. These processing conditions were selected to promote complete PVC fusion and the homogeneous incorporation of the plasticizer while avoiding excessive thermal degradation. The extrudates were then collected as continuous strips and cooled under ambient conditions until dimensional stability was achieved. Samples exhibiting visible defects, bubbles, phase separation, or pronounced surface irregularities were excluded from subsequent mechanical testing. The homogeneous regions of the extruded strips were used for specimen preparation. Mechanical test specimens were prepared from the homogeneous extruded PVC strips using a standardized dumbbell-shaped cutting die. Specimen geometry was selected in accordance with ASTM D638 [29]. However, prior to mechanical testing, the thickness of each specimen was measured at three different positions using a digital micrometer, and the average value was used for the calculation of tensile properties.

#### 2.4.2. Tensile Testing

The tensile properties of the plasticized PVC specimens were determined using an Instron 3345 universal testing machine (Instron, Norwood, MA, USA) in accordance with ASTM D638. Testing was performed using a 2 kN load cell at a crosshead speed of 50 mm min^−1^. The initial gauge length was 25 mm, and the specimens had an average thickness of 1.5 mm and width of 6 mm in the gauge section. Each formulation was tested using *n* = 5 independently prepared specimens. The following tensile parameters were determined from the resulting stress–strain curves: tensile strength (MPa), elongation at break (%), and elastic modulus at 100% elongation (MPa). Tensile strength was calculated from the maximum force divided by the initial cross-sectional area of the specimen, while elongation at break was calculated from the change in gauge length at failure relative to the initial gauge length. The modulus was determined according to the secant modulus at 100% elongation directly evaluated from the stress–strain curves suing Bluehill software (version v4.47, Instron, Norwood, MA, USA).

#### 2.4.3. Shore a Hardness Testing

The hardness of the plasticized PVC specimens was measured using a Shore A durometer (Model 3115, ZwickRoell GmbH, Ulm, Germany) according to ASTM D2240 [30]. Measurements were performed on flat, homogeneous regions of the specimens with sufficient thickness to minimize substrate-related effects. Before measurement, samples were conditioned under the standard conditions for 48 h. The durometer was positioned perpendicular to the specimen surface, and the indenter was brought into firm contact with the material without impact. The Shore A value was recorded after 15 s of contact.

#### 2.4.4. Accelerated Thermal Aging Test of Plasticized PVC

Accelerated thermal aging was performed to comparatively evaluate the short-term thermal aging behavior of PVC plasticized with commercial DOTP and PET-derived DOTP-TKT. PVC specimens prepared using the same formulations employed for the mechanical tests (100 phr PVC and 50 phr plasticizer) were placed in a laboratory oven maintained at 180 °C and exposed for a total of 6 h. Specimens were visually examined and photographed before thermal exposure (0 min) and after (60, 180, and 360 min) aging under identical observation conditions. Changes in specimen appearance, particularly discoloration and visible surface deterioration, were comparatively evaluated as indicators of heat-induced degradation. Commercial DOTP-plasticized PVC was used as the reference formulation, while PVC plasticized with DOTP-TKT was evaluated under identical conditions.

### 2.5. Statistical Analysis

Statistical analyses were performed using OriginPro software (version v9.0, OriginLab Corporation, Northampton, MA, USA). Unless otherwise specified, all experiments were independently conducted in triplicate (*n* = 3), and quantitative data are presented as the mean ± standard deviation (SD). Statistical differences between experimental groups were evaluated using the appropriate statistical tests, with differences considered statistically significant at *p* < 0.05.

## 3. Results and Discussions

### 3.1. Synthesis of Recycled DOTP

**Figure 3:**
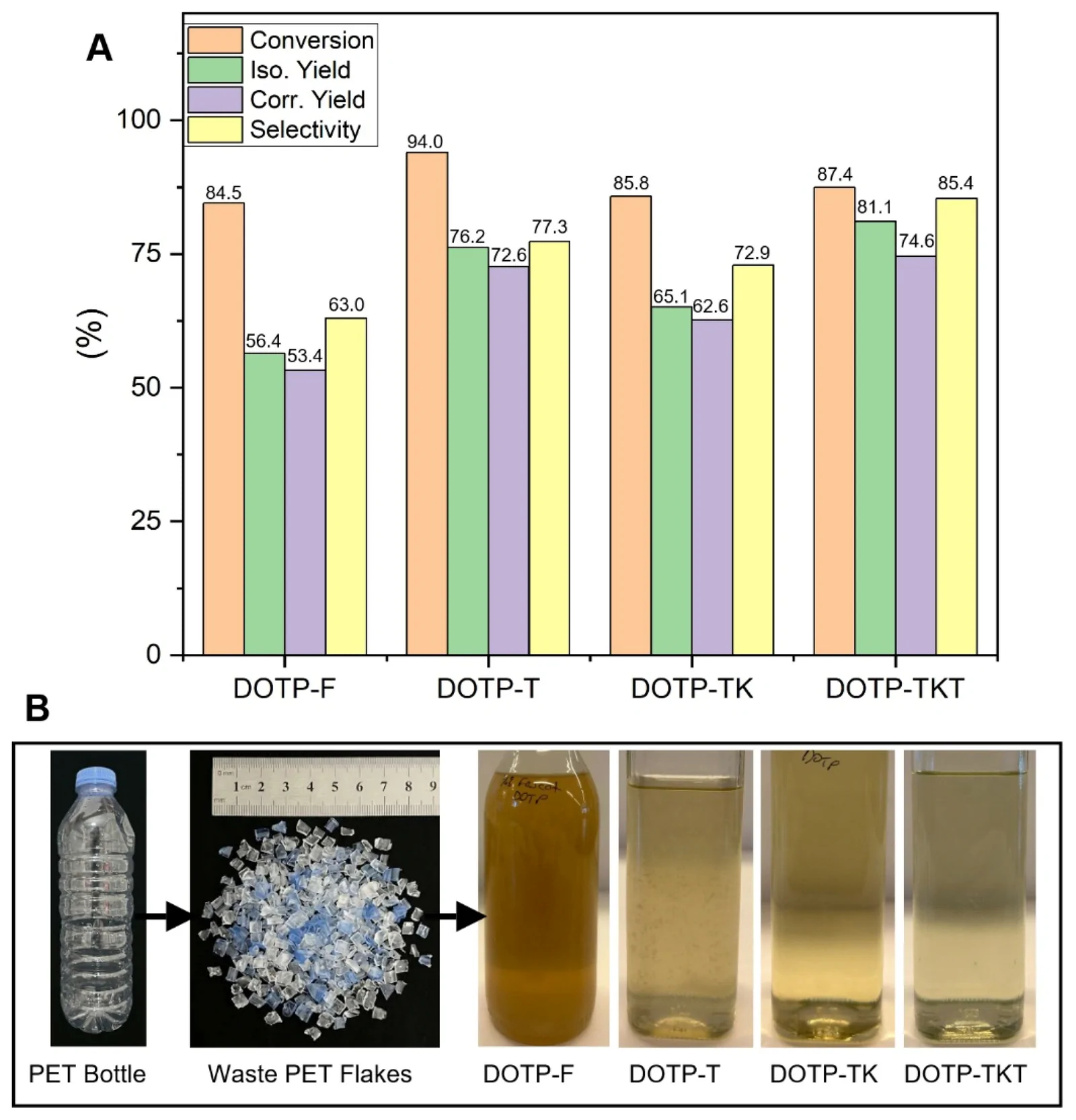
(**A**) Conversion, isolated yields, corrected yields and selectivity values obtained for synthesized DOTP samples prepared using different catalyst systems (DOTP-F, DOTP-T, DOTP-TK, and DOTP-TKT). (**B**) Schematic representation of conversion of waste PET bottles into DOTP plasticizers. Post-consumer PET bottles were mechanically processed into PET flakes and subsequently depolymerized and esterified to produce DOTP-F, DOTP-T, DOTP-TK, and DOTP-TKT.

The influence of the organometallic catalyst systems (F, T, TK, and TKT) on PET conversion, DOTP yield, and reaction selectivity during the alcoholysis/transesterification of waste PET with 2-EH is presented in Figure 3. All catalysts promoted substantial PET conversion (84–94%), confirming their effectiveness toward ester-bond cleavage. The T-catalyzed system exhibited the highest conversion (~94%), followed by TKT (~88%), TK (~86%), and F (~84%). The superior activity of T can be attributed to the strong Lewis acidity of titanium centers, which facilitates coordination with PET carbonyl oxygen, increases carbonyl electrophilicity, and promotes nucleophilic attack by 2-EH [20,31]. As shown in Figure 3, the catalyst systems depicted distinct effects on PET conversion and DOTP formation. The titanium catalyst (T) provided the highest PET conversion (~94%), whereas the hybrid TKT system showed a somewhat lower conversion (~88%). The gravimetrically determined isolated product yields were approximately 56.4%, 76.2%, 65.1%, and 81.1% for F, T, TK, and TKT, respectively. After correction using the relative DOTP contents determined independently by GC-FID, the corrected DOTP yields were approximately 53.2%, 72.6%, 62.6%, and 74.6% for F, T, TK, and TKT, respectively. This behavior was further reflected in DOTP selectivity, which increased from approximately 63.0% for DOTP-F to 72.9% for DOTP-TK and 77.3% for DOTP-T, reaching a maximum of 85.4% for DOTP-TKT. The comparatively poor selectivity of F and TK suggests the less efficient conversion of depolymerization intermediates into DOTP and greater persistence of oligomeric species, consistent with the slower development of catalytically active organotin species reported at elevated temperatures [15,32]. Such residual intermediates may also contribute to product losses during purification and particle formation upon storage. Moreover, the TKT system demonstrated the most favorable balance between PET conversion, product yields, and apparent selectivity among the investigated catalyst formulations. In particular, despite not providing the highest PET conversion, the hybrid catalyst resulted in a greater recovery of the targeted product and a higher apparent selectivity than the individually applied catalyst systems. These findings indicate a beneficial complementary effect arising from the combined use of the titanium and organotin catalysts under the investigated conditions. Titanium- and organotin-based catalysts are known to exhibit different catalytic characteristics in esterification and transesterification reactions [33,34]; therefore, their combination may provide a reaction environment that favors the conversion of PET-derived intermediates toward the targeted diester. However, the present endpoint experiments do not allow the individual contributions of the Ti and Sn components or the existence of a true synergistic interaction to be established. Accordingly, the improved performance of TKT is interpreted here as a complementary catalytic effect. This cooperative behavior provides a more favorable balance between polymer conversion and target product formation than either catalyst alone, consistent with the reported benefits of mixed-metal catalytic environments in transesterification reactions [18]. Consequently, the TKT system achieved the best overall reaction performance, combining high DOTP recovery with exceptional selectivity and improved product stability, highlighting its potential for efficient waste-PET-to-plasticizer upcycling.

Furthermore, the physicochemical properties of the PET-derived plasticizers were evaluated against commercial DOTP to assess their potential suitability for industrial application (Table 2). The acid value varied markedly with catalyst type. DOTP-T exhibited the lowest value (0.07 mg KOH g^−1^), satisfying the recommended commercial limit of <0.10 mg KOH g^−1^ [14], followed by DOTP-TKT (0.17 mg KOH g^−1^). Conversely, DOTP-TK and DOTP-F exhibited substantially higher values of 1.58 and 2.73 mg KOH g^−1^, respectively, suggesting the greater persistence of acidic reaction-derived species or incomplete esterification. Dynamic viscosity ranged from 124 to 250 cP, exceeding that of commercial DOTP (81 cP). DOTP-TK displayed the highest viscosity (250 cP), consistent with the possible presence of higher-molecular-weight oligomeric species [20]. In contrast, DOTP-TKT exhibited the lowest viscosity among the PET-derived products (124 cP), indicating the more effective conversion of oligomeric intermediates into lower-molecular-weight ester products. Catalyst selection also strongly influenced product color. APHA values ranged from 126 Pt–Co for DOTP-TKT to 243 Pt–Co for DOTP-F, compared with 17 Pt–Co for commercial DOTP. The residual coloration likely originates from post-consumer PET impurities, catalyst-derived species, and thermally induced side reactions [35]. All synthesized samples exhibited comparable densities (0.95–0.96 g cm^−3^), approaching the commercial reference (0.98 g cm^−3^), with minor deviations that may reflect residual 2-EH or other low-molecular-weight components [20]. İn summary, DOTP-TKT provided the most balanced physicochemical profile, combining relatively low acidity, viscosity, and coloration with a density close to commercial DOTP. Together with its higher synthesis yield and selectivity, these findings further support the beneficial role of hybrid titanium–organotin catalysis in improving PET-derived DOTP quality.

As summarized in Table 3, the reported efficiency of PET alcoholysis toward DOTP varies considerably depending on the catalyst system and reaction conditions. High DOTP yields have been achieved using ionic-liquid-assisted systems and deep eutectic solvents; however, direct comparisons solely on the basis of DOTP yield are complicated by substantial variations in catalyst loading, alcohol-to-PET ratios, reaction times, temperatures, and the use of auxiliary solvents or specialized reaction media. For instance, a Zn(OAc)_2_/[Bmim]Cl system afforded a 93.1% DOTP yield after 5 h at reflux, whereas a ChCl/Zn(OAc)_2_ deep eutectic solvent achieved an 84.7% yield with complete PET conversion within 1 h at 180 °C [36]. Alternatively, subcritical 2-EH alcoholysis attained a higher DOTP yield of approximately 97%, albeit under an extreme operating temperature of 300 °C.

In comparison, the hybrid TKT system evaluated herein achieved an isolated product yield of 81.1% (corresponding to an estimated GC-FID assay-corrected DOTP yield of 74.6%) at 220 °C for 4 h without requiring ionic liquids, NMP, or other auxiliary cosolvents. Although this value does not match the upper bound of literature yields, operating under neat, cosolvent-free conditions at a comparatively moderate temperature underscores the practical and catalytic feasibility of the combined Ti–Sn approach.

### 3.2. FT-IR and ^1^H-NMR Characterization of PET-Derived DOTP

**Figure 4:**
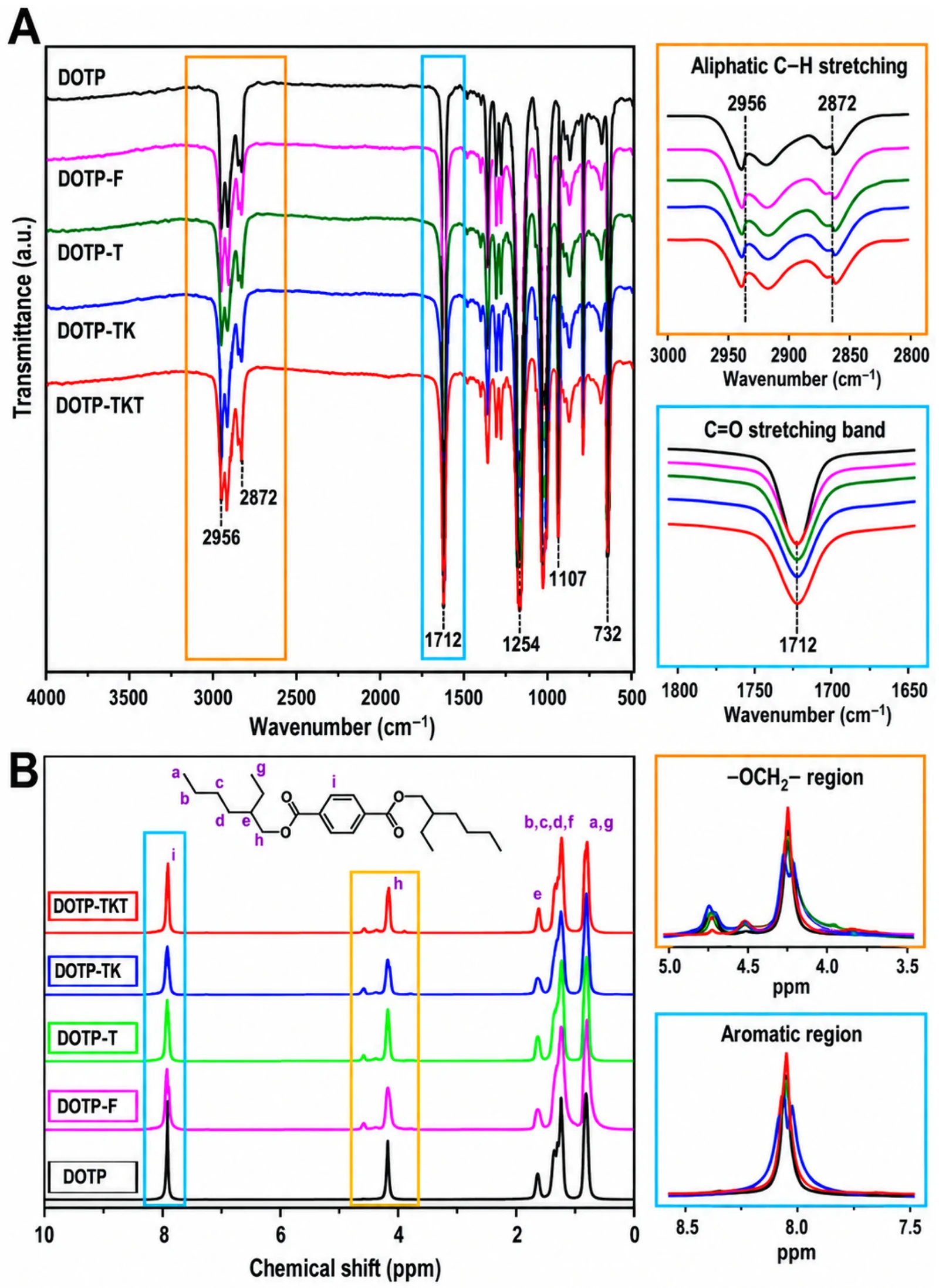
(**A**) FTIR spectra and (**B**) ^1^H-NMR spectra of commercial DOTP and DOTP samples synthesized from waste PET (DOTP-F, DOTP-T, DOTP-TK, and DOTP-TKT).

FTIR spectroscopy was employed to verify the chemical transformation of waste PET into DOTP and to assess catalyst-dependent structural differences relative to commercial DOTP (Figure 4A). All synthesized samples exhibited the characteristic vibrational features of the terephthalate diester structure, confirming successful alcoholysis/transesterification while preserving the aromatic terephthalate framework. The absence of a pronounced O–H stretching band at 3500–3300 cm^−1^ suggests the effective removal of residual ethylene glycol and excess 2-ethylhexanol during purification, although trace hydroxyl-containing intermediates cannot be excluded solely from FTIR analysis [32]. The prominent bands at approximately 2956 and 2872 cm^−1^ are assigned to the asymmetric and symmetric aliphatic C–H stretching vibrations of the introduced 2-ethylhexyl groups, respectively [38]. These bands were particularly well aligned between DOTP-TKT and commercial DOTP, supporting the efficient incorporation of the 2-ethylhexyl chains. Most importantly, an intense ester carbonyl stretching band at 1712 cm^−1^ was observed across all samples. The close correspondence of this band with commercial DOTP confirms the preservation of the terephthalate ester functionality following PET depolymerization and re-esterification [39]. In addition, characteristic bands at 1254 and 1107 cm^−1^ are attributable predominantly to ester C–O/C–O–C stretching vibrations, whereas the band at approximately 732 cm^−1^ is associated with the out-of-plane C–H deformation of the para-disubstituted aromatic terephthalate ring. Collectively, these features provide strong spectroscopic evidence for the formation of the targeted DOTP structure. Minor differences in band intensity and profile were nevertheless evident among the catalyst systems, reflecting variations in their local molecular environments and residual reaction-derived species [20]. In all, DOTP-TKT exhibited the closest spectral correspondence to commercial DOTP, with well-defined characteristic bands and minimal broadening. Combined with the GC-FID/GPC and physicochemical results, the FTIR findings support the ability of the hybrid titanium–organotin catalyst to favor the formation of a structurally homogeneous DOTP product while limiting incompletely converted PET-derived intermediates.

Figure 4B further elucidates the molecular structures of the PET-derived DOTP samples by ^1^H-NMR spectroscopy and compared with commercial DOTP. All synthesized products displayed the characteristic resonances of DOTP, confirming the successful conversion of the PET-derived terephthalate framework into the targeted di(2-ethylhexyl) ester. A sharp singlet at δ ≈ 8.0–8.1 ppm was assigned to the four chemically equivalent aromatic protons (i) of the para-disubstituted terephthalate ring [18]. The well-defined signal observed particularly for DOTP-T and DOTP-TKT indicates the preservation of the aromatic terephthalate structure. The characteristic resonance at δ ≈ 4.1–4.3 ppm corresponds to the ester-linked methylene protons (–OCH_2_–, h) of the 2-ethylhexyl groups, providing direct evidence for successful transesterification [39]. In addition, the weak resonances in the oxygenated-proton region provided evidence of residual intermediates. The shoulder near δ ≈ 4.65 ppm, more pronounced for DOTP-F and DOTP-TK, is attributed to ethylene-glycol-containing oligomeric species, consistent with their higher oligomeric fractions observed by GPC. Minor signals at approximately δ ≈ 4.4 and 3.9 ppm in DOTP-TKT may originate from ethylene-glycol-containing intermediates, including bis(2-hydroxyethyl) terephthalate and 2-ethylhexyl 2-hydroxyethyl terephthalate, consistent with the GC-FID results. In the aliphatic region, resonances at δ ≈ 1.2–1.7 ppm were assigned to the methine and methylene protons of the 2-ethylhexyl chains, whereas signals at δ ≈ 0.8–1.0 ppm correspond to terminal methyl groups [15]. When compared with commercial DOTP, their close agreement further confirms the formation of the expected molecular structure. In general, DOTP-TKT exhibited the closest spectral correspondence to commercial DOTP, with fewer prominent signals attributable to residual oligomeric species. Together with GPC and GC-FID analyses, the ^1^H-NMR results demonstrate that hybrid titanium–organotin catalysis promotes the efficient conversion of PET-derived intermediates toward a comparatively homogeneous DOTP product.

### 3.3. GC-FID Composition and Comparative GPC Assessment of Residual Oligomeric Species

**Figure 5:**
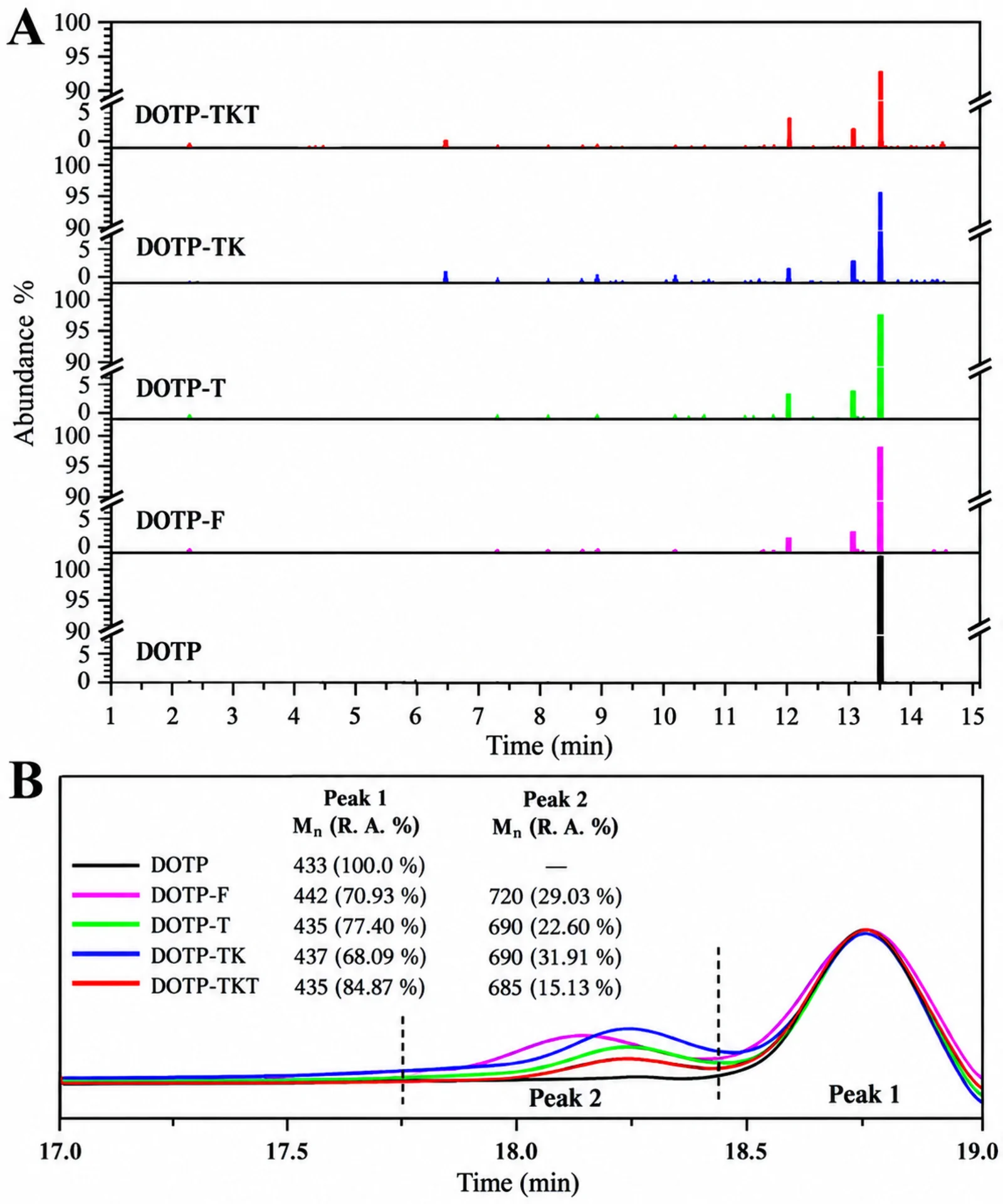
Chromatographic characterization of commercial and PET-derived DOTP products. (**A**) GC-FID chromatograms of commercial DOTP and PET-derived products obtained using different catalyst systems. (**B**) Comparative GPC profiles showing principal low-molecular-weight DOTP-associated fraction (Peak 1) and earlier-eluting higher-hydrodynamic-volume fraction (Peak 2). (R.A. = Relative Area).

GC-FID and GPC were used as complementary techniques to evaluate the chromatographic composition and molecular size distribution of commercial DOTP and PET-derived products (Figure 5A,B). Because these techniques rely on fundamentally different separation principles and analytical windows, their results were interpreted independently. GC-FID revealed a dominant DOTP-associated peak at approximately 13.5 min (Figure 5A), accounting for 99.5, 94.3, 95.3, 96.1, and 92.0% of the integrated chromatographic area for commercial DOTP, DOTP-F, DOTP-T, DOTP-TK, and DOTP-TKT, respectively. Minor signals between approximately 12.0 and 13.2 min were additionally detected in the PET-derived samples. These peaks correspond to intermediates generated during the PET alcoholysis reaction [20]; however, their definitive structures could not be verified due to the lack of reference standards or GC-MS confirmation [32]. Accordingly, the GC-FID data are reported as relative chromatographic composition rather than absolute chemical purity. On the other hand, GPC provided complementary evidence of catalyst-dependent differences in molecular size distributions (Figure 5B). Commercial DOTP exhibited a predominant low-molecular-weight region with an apparent Mn of ~433 g mol^−1^. Comparable principal regions (Peak 1) were observed for DOTP-F, DOTP-T, DOTP-TK, and DOTP-TKT, with apparent Mn values of ~442, 435, 437, and 435 g mol^−1^, respectively. The PET-derived samples additionally exhibited an earlier-eluting, higher-hydrodynamic-volume region (Peak 2), with apparent Mn values of ~720, 690, 690, and 685 g mol^−1^, respectively. Peak 2 represented 29.03, 22.60, 31.91, and 15.13% of the integrated GPC relative areas (R.A.), whereas Peak 1 accounted for 70.93, 77.40, 68.09, and 84.87%, respectively. Thus, DOTP-TKT exhibited the lowest relative contribution of higher-molecular-weight species at the 4 h reaction endpoint. These GPC results should be regarded as comparative and semi-quantitative because calibration employed polystyrene standards (1.2–400 kDa), whereas DOTP (390.56 g mol^−1^) lies below the validated calibration range. Collectively, GC-FID confirms DOTP as the predominant detected component, while GPC indicates that TKT produced the lowest relative higher-molecular-weight fraction under the investigated conditions.

### 3.4. Thermal Stability of PET-Derived DOTP

**Figure 6:**
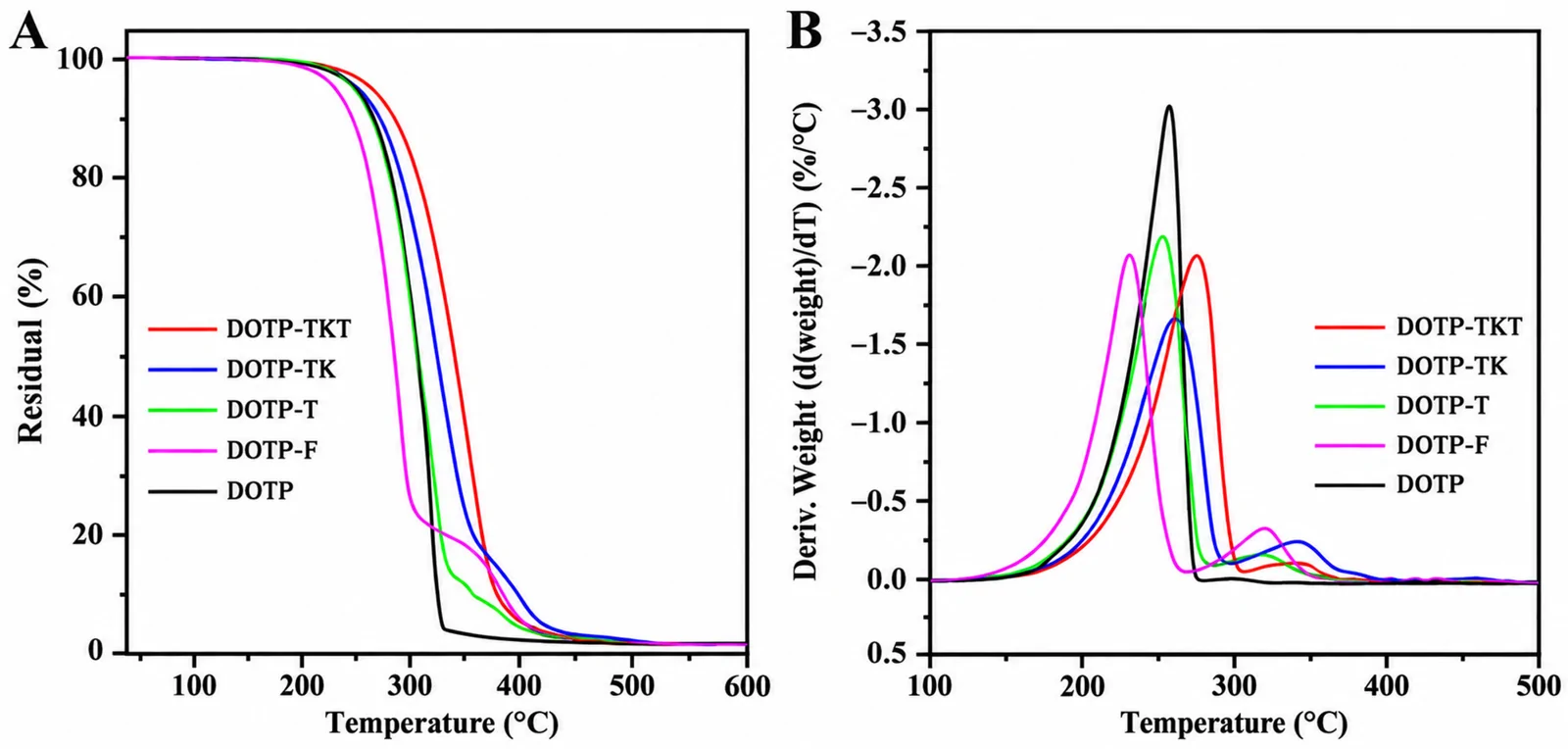
(**A**) TGA and (**B**) DTG curves of commercial DOTP and DOTP samples synthesized from waste PET (DOTP-F, DOTP-T, DOTP-TK, and DOTP-TKT).

The thermal stability and degradation behavior of the PET-derived DOTP samples were evaluated by TGA and DTG and compared with commercial DOTP (Figure 6). All samples exhibited their principal mass loss phases between approximately 200 and 400 °C, characteristic of the volatilization and thermal decomposition of low-molecular-weight terephthalate ester plasticizers [15,18]. Nevertheless, distinct catalyst-dependent differences were evident in the onset and maximum degradation temperatures and in the occurrence of secondary degradation stages. The commercial DOTP displayed predominantly single-step degradation, with decomposition initiating at ~210 °C and a DTG maximum (T_max_) near 275 °C. Its relatively narrow degradation profile is consistent with the high molecular homogeneity indicated by chromatographic analyses [20]. Among the PET-derived products, DOTP-TKT exhibited the most favorable thermal behavior, with decomposition beginning at ~210 °C and a principal T_max_ of ~283.5 °C. DOTP-TK showed similarly enhanced thermal resistance, also reaching a principal T_max_ of ~283.5 °C. In comparison, DOTP-T degraded up to approximately 278–279 °C, suggesting that the enhanced stability of TKT is primarily associated with the contribution of the organotin component rather than the titanium catalyst alone [6]. DOTP-F exhibited the lowest thermal stability, with degradation occurring approximately between 200 and 265 °C and T_max_ near 253 °C. The PET-derived samples additionally exhibited higher-temperature DTG features at approximately 313.0, 313.5, 331.0, and 332.0 °C for DOTP-F, DOTP-T, DOTP-TK, and DOTP-TKT, respectively. These secondary events are consistent with the decomposition of less volatile, higher-molecular-weight species, including residual oligomeric fractions identified by GPC. Importantly, their relative contribution, rather than merely their decomposition temperature, reflects oligomer abundance of which this contribution was the greatest for DOTP-TK and the lowest for DOTP-TKT, consistent with the GPC-derived molecular distributions. The recovered residual mass above 500 °C remained below ~1% for all samples, indicating negligible non-volatile inorganic residues. In summary, the hybrid TKT catalyst provided the best balance between molecular homogeneity and thermal resistance. Moreover, the principal degradation of all the PET-derived DOTP samples occurred well above typical PVC processing temperatures (170–180 °C), supporting their thermal suitability for PVC plasticization.

### 3.5. Tensile, Hardness and Thermal Aging Performance of DOTP

**Figure 7:**
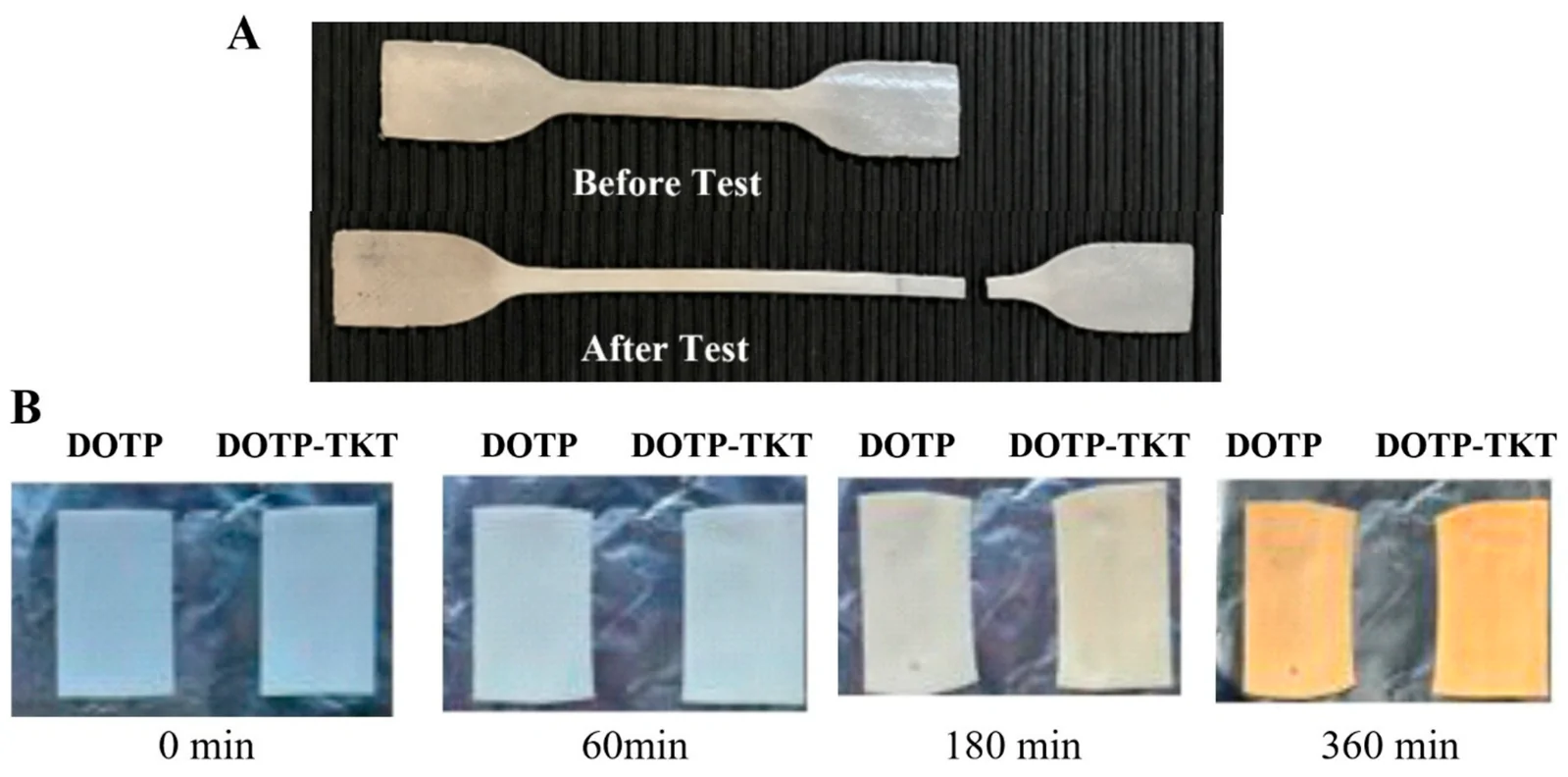
(**A**) An image showing the PVC-based materials prepared with commercial DOTP and synthesized DOTP-TKT before and after tensile testing. (**B**) A visual comparison of the accelerated thermal aging behavior of PVC specimens plasticized with commercial DOTP and PET-derived DOTP-TKT. Specimens were maintained at 180 °C for 6 h and photographed before aging (0 min) and after 60, 180, and 360 min of thermal exposure.

The plasticization performance of PET-derived DOTP-TKT was evaluated in flexible PVC and benchmarked against commercial DOTP using Shore A hardness, tensile strength, elongation at break, and elastic modulus (Table 4). In general, DOTP-TKT produced a favorable combination of strength, stiffness, and ductility, demonstrating its suitability as a functional PVC plasticizer. In addition, PVC plasticized with DOTP-TKT exhibited a Shore A hardness of 91.50 ± 0.57 compared with 89.25 ± 0.50 for commercial DOTP, corresponding to a modest increase of ~2.5%. This slightly higher hardness suggests somewhat reduced plasticization efficiency at equivalent loading, potentially arising from residual higher-molecular-weight species from the polymer. Nevertheless, both formulations retained the characteristics of flexible PVC. More importantly, DOTP-TKT significantly improved tensile performance. As observed from the deduced results, tensile strength increased from 22.22 ± 1.96 MPa for commercial DOTP to 24.79 ± 0.53 MPa for DOTP-TKT, representing an improvement of ~11.6%. The elastic modulus similarly increased from 32.51 ± 7.18 to 37.42 ± 8.91 MPa (~15.1%), indicating enhanced resistance to deformation and potentially more effective stress transfer within the plasticized PVC matrix. This improvement may be associated with residual PET-derived oligomeric species remaining, which may contribute as secondary plasticizers within the PVC matrix, in DOTP-TKT after the depolymerization process. Moreover, the increased strength and stiffness were not accompanied by reduced ductility. Elongation at break increased from 409.1 ± 32.8% for commercial DOTP to 434.9 ± 5.3% for DOTP-TKT (~6.3%). The substantially lower variability observed for DOTP-TKT further suggests more reproducible deformation behavior, potentially reflecting homogeneous plasticizer distribution throughout the PVC matrix. Thus, DOTP-TKT simultaneously enhanced tensile strength and extensibility rather than producing the conventional strength–ductility trade-off. An image representation of the material before and after tensile testing is shown in Figure 7A. Collectively, the obtained results demonstrate that PET-derived DOTP-TKT provides mechanical performance comparable to, and for the measured tensile properties exceeding, that of commercial DOTP. However, further migration, aging, and long-term durability studies would be required to establish suitability for application-specific commercial use.

Thermal aging is a critical consideration for plasticized PVC, as prolonged exposure to elevated temperatures promotes dehydrochlorination, progressive degradation, and discoloration; consequently, oven-aging protocols are widely employed to compare the thermal stability of PVC formulations [21]. Accordingly, the accelerated thermal aging behavior of PVC plasticized with commercial DOTP and PET-derived DOTP-TKT was comparatively evaluated at 180 °C for up to 360 min (Figure 7B). Prior to thermal exposure (0 min), both formulations exhibited a comparable light-colored appearance. After 60 min of heating, no pronounced visual changes were observed in either sample. With extended exposure time, both formulations underwent progressive discoloration, shifting toward a distinct yellow–orange hue after 180 min and becoming pronounced at 360 min, which aligns with the typical thermal degradation pathway of PVC matrices. Importantly, the DOTP-TKT-plasticized specimens followed an aging trajectory essentially identical to that of the commercial DOTP reference throughout the test period, showing no significant qualitative divergence in color stability. These visual observations demonstrate that substituting commercial DOTP with PET-derived DOTP-TKT does not compromise the short-term thermal aging resistance of the plasticized PVC formulations.

### 3.6. Limitations and Future Research Perspectives

Although the hybrid titanium–organotin catalyst demonstrated promising selectivity, product quality, thermal stability, and PVC plasticization performance, several limitations should be addressed before industrial implementation. The present study was conducted at laboratory scale using a single post-consumer PET feedstock and fixed reaction conditions; therefore, the influence of feedstock heterogeneity, contamination, catalyst loading, PET/2-ethylhexanol ratio, temperature, and reaction time requires systematic optimization. Moreover, while complementary catalytic behavior was observed for the TKT system, elucidating whether this effect originates from a genuine Ti–Sn synergism necessitates time-resolved kinetic analyses alongside matched catalyst loadings (e.g., individual versus combined systems at equivalent total molar loadings) and varied Ti/Sn ratios under identical conditions.

A further critical consideration concerns the organotin component within the hybrid system. Although combining the titanium catalyst with TK halved the organotin requirement relative to the TK-only formulation, catalyst recovery and reusability remained outside the scope of this work. Crucially, the residual tin concentration, organotin speciation in the purified DOTP, and the potential migration or leaching of Sn species from the plasticized PVC matrix were not quantified. Consequently, the present findings establish catalytic feasibility and material performance rather than regulatory or toxicological compliance. To facilitate commercial translation, subsequent investigations should quantify residual tin levels via ICP-OES or ICP-MS, explore strategies for catalyst recycling or complete organotin phase-out, and comprehensively evaluate long-term migration, weathering, and durability in PVC applications. Finally, life-cycle assessment and techno-economic analysis are required to quantify the environmental and economic advantages of the proposed PET-to-DOTP route relative to conventional petrochemical DOTP production and alternative PET recycling technologies.

## 4. Conclusions

This study demonstrates the selective upcycling of post-consumer PET into DOTP through a hybrid titanium–organotin catalytic strategy. Comparative evaluation under identical reaction conditions established that catalyst selection governs not only PET depolymerization but also target product selectivity, molecular homogeneity, thermal stability, and end-use plasticization performance. Although titanium(IV) isopropoxide afforded the highest PET conversion (94%), the hybrid TKT system achieved the best overall performance, providing the highest DOTP yield (81%) and selectivity (~92%). This distinction highlights that the efficient conversion of PET-derived intermediates into the target diester is more critical to product quality than maximizing polymer conversion alone. The TKT system provided the most favorable balance between product yield and apparent selectivity among the investigated catalyst formulations. GC-FID and GPC provided complementary but independent information on product composition: GC-FID indicated the relative DOTP content of the recovered products, whereas comparative GPC profiles revealed differences in the occurrence of residual higher-molecular-weight species. These results were therefore interpreted independently rather than combined into a single purity metric. FTIR and ^1^H-NMR confirmed the formation of the targeted terephthalate diester structure, while TGA demonstrated favorable thermal stability. DOTP-TKT further exhibited a relatively low acid value (0.17 mg KOH g^−1^), viscosity (124 cP), and APHA color (126 Pt–Co). Importantly, PVC plasticized with DOTP-TKT exhibited an increase in tensile strength, elastic modulus, and elongation at break, compared with commercial DOTP. In summary, the complementary catalytic behavior of titanium and organotin species provides an effective route for directing waste PET depolymerization toward value-added DOTP. These findings establish hybrid organometallic catalysis as a promising strategy for PET-to-plasticizer upcycling and support further process optimization, scale-up, catalyst recovery, and long-term PVC performance assessment.

## Figures and Tables

**Table 1:** Weights of reactants and catalyst used in DOTP synthesis.

Code	PET	2-EH	Catalyst
DOTP	-	-	-
DOTP-F	192 g (1 mole)	520 g (4 moles)	5.76 g F
DOTP-T	192 g (1 mole)	520 g (4 moles)	5.76 g T
DOTP-TK	192 g (1 mole)	520 g (4 moles)	5.76 g TK
DOTP-TKT	192 g (1 mole)	520 g (4 moles)	2.88 g TK + 2.88 g T

**Table 2:** Physicochemical properties of DOTP samples synthesized with different catalyst systems and commercial reference.

Code	Viscosity (20°) (cp)	Density (20°) (g/cm^3^)	Acid Number (mgKOH/g)	Color (Pt–Co)
DOTP-F	200	0.96	2.73	243
DOTP-T	158	0.96	0.07	160
DOTP-TK	250	0.95	1.58	228
DOTP-TKT	124	0.95	0.17	126
DOTP	81	0.98	0.01	17

**Table 3:** Comparison of catalytic systems reported for alcoholysis of PET to DOTP using 2-ethylhexanol.

Catalytic System	Catalyst Loading	EH/PET Ratio	Temp. (°C)	Time (h)	PET Conversion (%)	DOTP Yield (%)	Main Feature/Limitation	Ref.
Tetrabutyl titanate (TBT) + NMP	0.5 wt%	4:1 mol/mol	Not explicitly stated in abstract	2	n.r.	78.1	NMP promotes PET dissolution and accelerates transition to homogeneous alcoholysis	Shi et al. [21]
Zn(OAc)_2_ + [Bmim]Cl cosolvent	1.2 wt%	2-EH/PET = 2:1 wt/wt	190–200	5	~100	93.1	High conversion/yield; requires ionic-liquid cosolvent	Chen et al. [35]
ChCl/Zn(OAc)_2_ DES	5 wt%	5:1 mol/mol	180	1	100	84.7	Short reaction time and complete PET conversion; relatively high DES loading	Zhou et al. [24]
Sulfonic IL–ZnCl_2_	high catalyst loading	3.39-fold 2-EH excess	210	8	n.r.	95.7	High DOTP yield but long reaction time/high IL catalyst loading	Liu et al. [37]
Titanium butoxide	—	3.39-fold 2-EH excess	210	8	n.r.	89.0	Conventional transesterification catalyst; long reaction time	Liu et al. [37]
Sulfuric acid	—	3.39-fold 2-EH excess	210	8	70.1	67.5	Lower catalytic activity for PET transesterification	Liu et al. [37]
Subcritical 2-EH, no conventional catalyst	—	4:1 wt/wt	300	3	100	97.0	Very high conversion/yield but requires high-temperature subcritical conditions	Liu et al. [37]
T/TK hybrid (TKT), present study	3 wt% total (1.5% + 1.5%)	4:1 mol/mol	220	4	~88	~74.6	Moderate temperature; no IL/NMP cosolvent; complementary Ti–Sn system; residual Sn and catalyst recovery require further evaluation	Present work

**Table 4:** Mechanical performance of flexible PVC compounds prepared with commercial DOTP and synthesized DOTP-TKT.

Property		Flexible PVC Produced with	
		DOTP	DOTP-TKT
Hardness (Shore A)		89.25 ± 0.50	91.50 ± 0.57
Tensile Test	Tensile strength (MPa)	22.22 ± 1.96	24.79 ± 0.53
	Strain at break (%)	409.10 ± 32.80	434.90 ± 5.30
	Modulus of elasticity (MPa)	32.510 ± 7.18	37.42 ± 8.91

## Data Availability

The data presented in this study will be made available upon request.
